# Liposomal Formulations Enhance the Anti-Inflammatory Effect of Eicosapentaenoic Acid in HL60 Cells

**DOI:** 10.3390/pharmaceutics14030520

**Published:** 2022-02-26

**Authors:** Puneet Kaur, Jin Gao, Zhenjia Wang

**Affiliations:** Department of Pharmaceutical Sciences, College of Pharmacy and Pharmaceutical Sciences, Washington State University, Spokane, WA 99210, USA; p.kaur@wsu.edu (P.K.); jin.gao3@wsu.edu (J.G.)

**Keywords:** eicosapentaenoic acid, liposomes, anti-inflammation, reactive oxygen species

## Abstract

Dietary omega 3 polyunsaturated fatty acids (PUFAs), including docosahexaenoic acid (DHA) and eicosapentaenoic acid (EPA), have been reported to be beneficial for cardiovascular diseases and cancer. Such diseases share a common pathophysiological feature of inflammation responses, such as unbalanced oxidative stress and increased cytokine release. PUFAs show anti-inflammatory effects, and thus, they are potential therapeutics to treat inflammatory disorders. Here, we proposed a novel liposomal formulation of EPA (EPA-liposomes), and the liposome was PEGylated to increase their stability. In the study, we measured the physicochemical characteristics of EPA-liposomes and their anti-inflammatory effects in neutrophil-like cells (HL 60 cells). The results showed that EPA-liposomes dramatically decreased the production of NO, ROS, and cytokines compared to EPA alone, and the molecular mechanism is associated with biosynthesis of RvE1 from EPA, and RvE1 binds to GPCRs to mediate the anti-inflammatory effects.

## 1. Introduction

At present, polyunsaturated fatty acids (PUFAs), including an ω-3 series, are mainly used as dietary supplements. Meanwhile, it was found that these dietary PUFAs affect a wide variety of physiological processes, such as antiatherogenic [1], antiaggregatory [2], and anti-inflammatory [3] effects.

Lipid-based nanocarriers can be efficiently internalized by phagocytic cells and hence, are an attractive system to deliver PUFAs to immune cells. Macrophages and neutrophils are known to be the most efficient uptake of nanoparticles and may act as a reservoir for nanotherapeutics. The characteristics of these immune cells may facilitate the conversion of omega 3 polyunsaturated fatty acids to the cascade metabolites like resolving D1 (RvD1) [4,5], resolving E1 (RvE1) [6,7], the well-known pre-resolving mediators. However, the application of PUFAs is limited to diet due to their low transformation in vivo.

Modern nanotechnology provides a promising strategy for drug delivery because most drugs face challenges in delivery efficiency [8,9,10]. Liposomal formulations, well-studied nanoparticles [11,12], allow the solubilization of unsaturated fatty acids, such as eicosapentaenoic acid (EPA), in aqueous solutions at high concentrations. In addition, PEGylation will prolong the circulation time of liposomes and increase the chance for extravasation by EPR (enhanced permeability and retention) effects in inflamed tissues. Long-circulating PEGylated liposomes can also improve the chemical stability of EPA, which are susceptible to fast oxidation [13].

Previous studies found that DHA-trapped liposomes could significantly inhibit the production of nitric oxides (NO), reactive oxygen species (ROS), and cytokines in macrophage-like cells [14]. Neutrophils, as another main population of leukocytes, especially during inflammation, possess the enzymes that contribute to the metabolism of EPA [15,16]. Therefore, it may be possible to convert EPA into a series of cascading metabolites of EPA.

Here, a nanomedicine-based approach is presented for delivering effective levels of EPA to inflammatory cells (neutrophil like cells, HL 60 cells), and we tested the anti-inflammatory effects in vitro. The potential mechanism behind the observed phenomenon was also studied.

## 2. Materials and Methods

### 2.1. EPA Liposome Preparation

**Figure 1:**
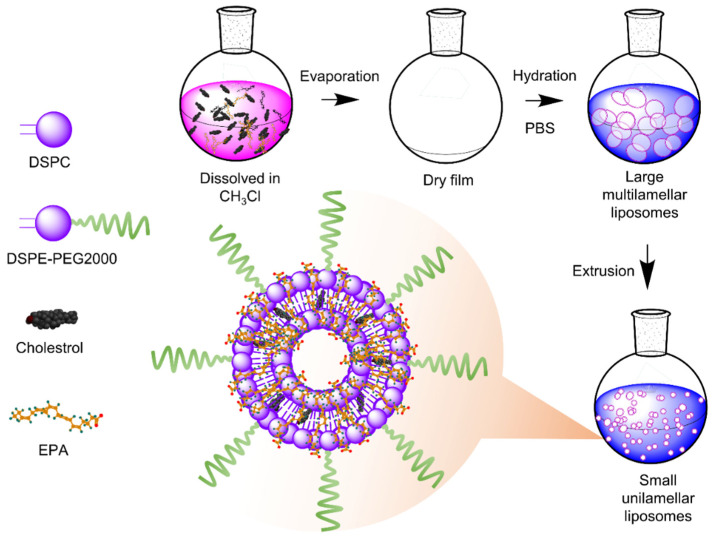
Liposomes were prepared by lipid film hydration method and EPA was efficiently loaded in a lipid bilayer of liposomes.

EPA loaded liposomes (EPA-liposomes) were prepared with DPPC, cholesterol, DSPE-PEG 2000, and EPA with a molar ratio of 1:1:1:0.15 using a lipid film hydration method. EPA was dissolved with cholesterol, DSPE-2000, and DPPC in a round bottom flask, and then this mixture was mixed for one hour on a rotating plate for the proper mixing, and a lipid layer was prepared using rotatory evaporation. After the lipid film was formed, the lipid film was hydrated with 5 mL of PBS pH 7.4 to form a lipid suspension. To downsize and to form a uniform size of liposomes, the lipid suspension was reduced by multiple sequential extrusion made of polycarbonate membranes with a final filter of 100 nm. The fluorescent dye-stained liposomes were prepared following the same steps, except the 0.2% (*w*/*w*) DiD (Invitrogen, Eugene, OR, USA) was supplemented in the lipid mixture. The preparation is illustrated in Figure 1.

### 2.2. Size and Zeta Potential Measurement

The mean particle-size distribution and polydispersity index (PDI) of liposomes were determined by dynamic light scattering (DLS) using a Malvern Zetasizer ZS-90 (Malvern Instruments, Malvern, UK) with a JDS Uniphase 22 mW He–Ne laser operating at 632 nm, an optical fiber-based detector and a digital LV/LSE-5003 correlator. All measurements were performed at a 90° angle. The zeta potential of the liposomes was determined by laser doppler electrophoresis using Zetasizer. Liposomes were diluted in fresh PBS (pH 7.4) prior to measurements.

### 2.3. Calculation of the Loading Efficiency and Encapsulation Efficiency of EPA

EPA-liposomes were sampled, and the trapped EPA was extracted with 9-fold volume of pure methanol. After 10 min, the mixture was centrifugated at 15,000× *g* for 10 min and the supernatant was analyzed using high performance liquid chromatography (HPLC).

Loading efficiency% = Amount of EPA loaded (mg)/Amount of total EPA loaded plus liposomes (mg) × 100%.

Encapsulation efficiency% = Amount of EPA loaded (mg)/Amount of total EPA feed (mg) × 100%.

### 2.4. Release Studies

First, 0.5 mL EPA-liposomes were placed into the Float-A-Lyzer G2 device (MWCO: 30 kD, Spectrum Laboratories, Dominguez, CA, USA). Then the devices were placed in 50 mL centrifugation tubes with 25 mL PBS each. The samples were taken 10 µL at 5 min, 10 min, 1, 2, 4, 8, 16, 24 h and stored at the −20 °C. Upon analysis with HPLC, the samples were extracted with 90 µL pure methanol and were centrifugated at 12,000× *g* for 5 min. The supernatant was subjected to analysis for EPA contents with HPLC.

### 2.5. EPA Measurement by HPLC

The EPA was quantified by an HPLC system (Alliance 2690, Waters, Worcester County, MA, USA) against a standard curve 100, 33, 11, 3.7, 1.2, 0.4, 0.13 μg/mL of EPA using acetonitrile as solvent. The HPLC was set in a mobile phase of acetonitrile:H_2_O = 70:30 at 1.3 mL/min. The column was Restek C8 150 × 4.6 mm. The EPA signal was monitored at 208 nm.

### 2.6. Stability Study

The EPA-liposomes or free EPA in PBS buffer (1 mg/mL) were placed in 1.5 mL centrifuge tubes and the tubes were stored at 4 °C. The size and the EPA concentrations were measured every week by DLS and HPLC, respectively. The experiments were performed in triplicate.

### 2.7. TEM Imaging

The liposomes in 5 µL were loaded on a grid and the morphology was imaged by transmission electron microscopy (FEI Tecnai G2 20 Twin TEM (Hillsboro, OR, USA).

### 2.8. Cell Culture

The HL60 cell line (ATCC, Manassas, VA, USA) was cultured in a complete growth medium prepared with RPMI 1640 (Corning, Manassas, VA, USA) + 10% FBS (Atlanta Biologicals, Flowery branch, GA, USA), and 1% antibiotics stocks (Life Technologies, Grand Island, NY, USA). Then, 1.25% (*v/v*) DMSO (Millipore-Sigma, St. Louis, MO, USA) was added into the media to differentiate for 4 days to become neutrophil-like cells.

### 2.9. Confocal Imaging

First, 1 × 10^5^ differentiated HL60 cells were incubated with 80 µg DiD-stained EPA-liposomes for 30 min at 37 °C in the RPMI1640 medium without FBS and then collected to prepare slides with a cytocentrifuge (Cytopro 7620, Wescor, Logan, UT, USA). Green cell plasma membrane staining dye (1:1000, Invitrogen, Eugene, OR, USA) was diluted to stain the cells for 15 min, and a drop of ProLong Gold^®^ Antifade reagent with DAPI (Invitrogen, Eugene, OR, USA) was added to the cells. A coverslip was applied on the slide. Four hours later, the cells were observed using a Nikon A1R+ confocal laser scanning microscope (Nikon, Japan).

### 2.10. Cell Viability Assessment

Cell viability was determined as described previously with slight revision [17]. Briefly, the differentiated HL60 cells were plated in 96-well plates at an initial density of 8000 cells/well in the presence of 100 ng/mL lipopolysaccharide (LPS, Millipore-Sigma, St. Louis, MO, USA) and EPA, in the form of free or liposomes, was supplemented to final concentrations of 40 and 80 µg/mL. The cells were further cultured in a humidified atmosphere containing 5% CO_2_. After incubation of 20 h, 10 μL of the cell proliferation reagent CCK-8 (Promega, Madison, WI, USA) was added in every experimental well. 4 h later, the absorption at 490 nm was measured by a microplate reader (SynergyNeo, BioTek, Winooski, VT, USA). All experiments were performed in hex plicate.

### 2.11. Inhibition of Production of Reactive NO and ROS

To evaluate the effect of liposomal formulations on the release of NO, differentiated HL60 cells were seeded at 100,000 cells per well in a 96-well plate in fresh medium supplemented with the respective treatments of EPA-liposomes or free EPA at the indicated concentrations in the presence of 100 ng/mL LPS. After 24 h of incubation, the cell-culture medium was removed pretreatment or in the pre-stimulation setup, and the supernatant was collected for a nitric oxide assay with Griess reagents (EnzoLife Sciences, Farmingdale, NY, USA) following the attached protocols. The NO levels were represented by the absorbance was measured at 550 nm on a plate reader. The collected cells were resuspended in a white 96-well plate for ROS measurement using a total ROS detection kit (Enzo Life Sciences, Farmingdale, NY, USA) per the guidance of the manual.

### 2.12. Cytokine Measurement

Differentiated HL60 was seeded at 2.5 × 10^5^ cells per well in a 24-well plate. EPA in either liposomal or free form was added to the predesigned concentrations. The medium was replaced by a medium containing 100 ng/mL LPS and cultured for an additional 22 h. The supernatant was collected and subjected to analysis for cytokines. TNF-α, IL-1β, and IL-6 were measured by enzyme-linked immunosorbent assay (ELISA) according to the manufacturer’s instructions (ELISA Max™ deluxe set, BioLegend, San Diego, CA, USA).

### 2.13. Metabolite Measurement

The HL60 cells were differentiated using 1.25% (*v/v*) DMSO for 4 days and then the 3 × 10^6^ cells were incubated with 0.25 mg/mL EPA in the form of free or liposomes for 8 h. The total cell cultures were diluted with 2-fold volume of cold ethanol. The mixtures were centrifugated at 10,000× *g* for 20 min and the supernatants were extracted with the same volume of chloroform. The upper water solution was discarded, and the lower chloroform was dried with nitrogen flow. Finally, the remnant was dissolved in 1 mL methanol for the measurement of RvE1 and 18-HEPE. The quantitation of RvE1 and 18-HDPE in cell culture medium was conducted on a UPLC-mass system (Shimadizu, Kyoto, Japan). To do so, 5 μL volume was injected for analysis. The UPLC-mass condition for 18-HEPE and RvE1 is as follows: the flow speed: 0.4 mL/min, and the column was Agilent BEH C18, 4.6 × 50, 1.8 µm. The mobile phase included water (A) and methanol (B), both supplemented with 10 mM ammonium acetate. The gradient: B increased from 90% to 100% in 2 min and maintained at 100% for 2 min. The ion pair for quantification was set at 317 > 215, or 349 > 107 for 18-HEPE and RvD1, respectively. A standard of SPM E-series LC-MS Mixture (Cayman, Ann Arbor, MI, USA) was used for identification and quantitation.

### 2.14. Statistics

The statistical analysis was performed using SPSS software 11.0 and the non-parametric one-way ANOVA. The differences between groups were considered significant at *p* ≤ 0.05, very significant at *p* ≤ 0.01, and the values were expressed as means ± SD.

## 3. Results

### 3.1. Preparation and Characterization of EPA-Liposomes

**Figure 2:**
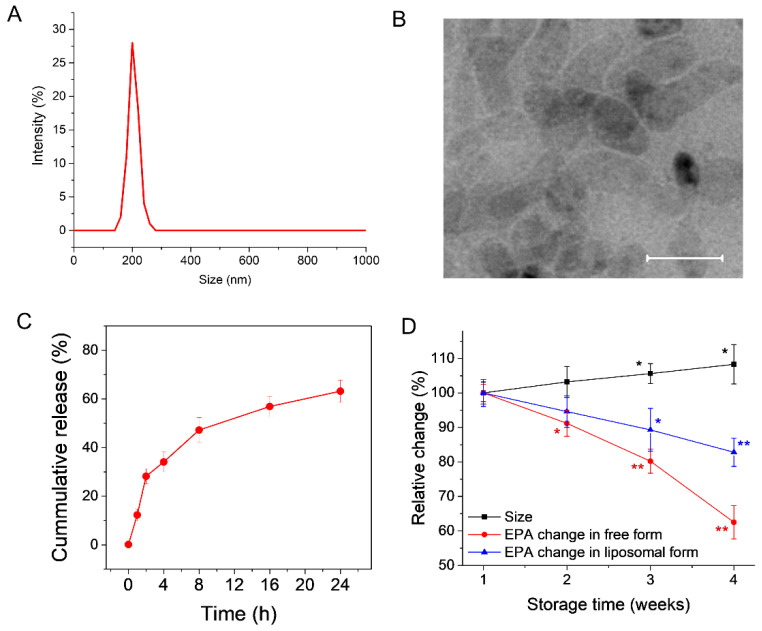
Characterization of EPA-liposomes. (**A**) Size distribution of the EPA-liposomes measured by DLS. (**B**) TEM imaging of the EPA-liposomes. Scale bar 200 nm. (**C**) The release profile of EPA from of the EPA-liposomes in PBS at 37 °C with shaking. (**D**) The change of size of the EPA-liposomes, the change of free EPA in PBS and the liposomal EPA in PBS over time when sored at 4 °C. * *p* < 0.05, ** *p* < 0.01 compared to the individual first measurement. *n* = 6 for size measurement. The data are expressed as means ± SD, *n* = 3 unless otherwise specified.

The EPA trapped liposomes were prepared using a lipid film hydration method and characterized for their size, zeta potential, polydispersity index, and entrapment efficiency. Using the DLS method, the size of the EPA-liposomes was determined to be 202 nm, with a PDI value of 0.17 (Figure 2A). When observed under a transmission electron microscope, the structure of the EPA-liposomes showed a spherical shape, with a bit of transformation when the dry grids for transmission electron microscopy (TEM) imaging were prepared, as observed in Figure 2B. The trapped EPA could be released gradually over time from EPA-liposomes upon being incubated at 37 °C (Figure 2C). The encapsulation efficiency of EPA was calculated to be 88.1%, and the loading efficiency of EPA was 14.3%. We also studied the stability of EPA-liposomes and found the size increased a little bit when the formulation was stored at 4 °C. The trapped EPA was also found degraded gradually but at a slower speed compared to the free EPA (Figure 2D), indicating the liposomal formulation may protect EPA from degradation caused by oxygen and light [18,19].

### 3.2. EPA-Liposomes Can Be Efficiently Internalized by HL60 Cells

**Figure 3:**
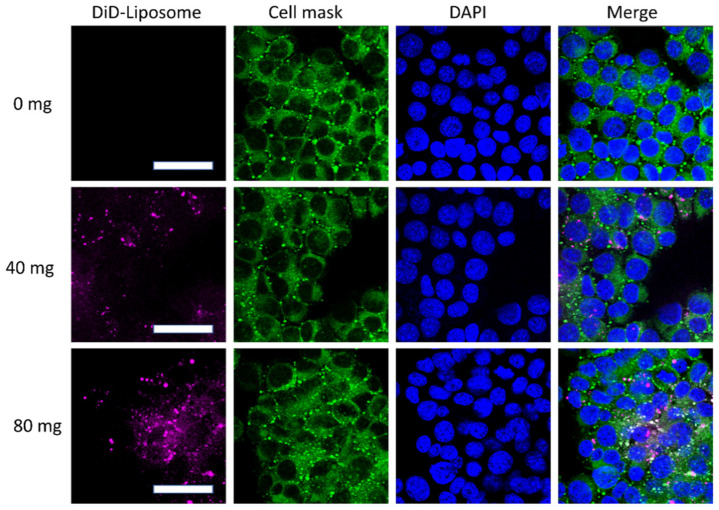
The internalization of the EPA-liposomes HL60 cells. The differentiated HL60 cells were treated with 100 ng/mL LPS for 3 h and incubated with DiD-stained EPA-liposomes for 30 min in the absence of FBS. Cell Mask green was used to cell membrane by incubating 15 min at room temperature. Scale bar was 50 µm.

Liposomes, as a mature and efficient drug delivery system, can be efficiently internalized by cells [14,20]. Therefore, EPA-liposomes may be an efficient system to deliver EPA in cells. Upon incubation with EPA-liposomes, differentiated HL60 bound to and internalized EPA-liposome in a liposome concentration-dependent manner, indicated by the increased fluorescent dye of the liposomes were colocalized with the cells (Figure 3).

### 3.3. EPA-Liposomes Increased the Cell Viability of Activated HL60 Cells

**Figure 4:**
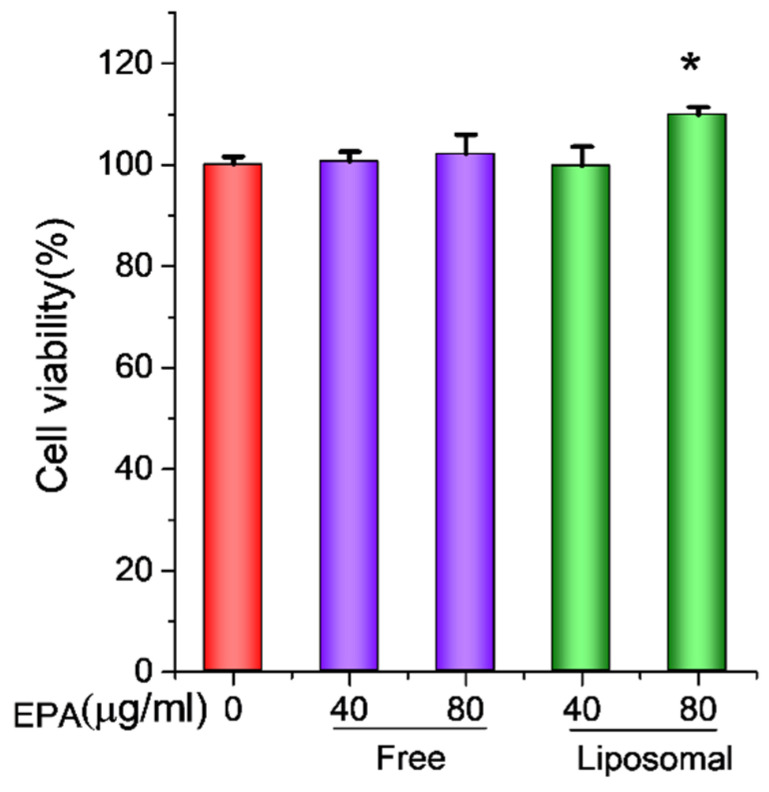
The cell viability enhancement by EPA-liposomes. The differentiated HL60 cells were treated with either free EPA or EPA-liposomes at the indicated concentrations in the presence of LPS (100 ng/mL) for 24 h and the cell viability was determined by the CCK-8 kit. The data are expressed as means ± SD. * *p* < 0.05, *n* = 6.

Before investigating the anti-inflammatory effect of EPA-liposomes, we firstly studied their effects on cell viability in HL 60 cells. To mimic neutrophils, we differentiated premature human acute promyelocytic leukemia cell line HL60 with 1.25% DMSO [5,21,22]. As shown in Figure 4, the differentiated HL60 cells did not show any increased cell viability when incubated with up to 80 µg/mL free EPA, while the liposomal EPA could significantly increase the cell viability. The results imply that EPA alone is not toxic to HL 60 cells. The cell viability under the liposomal formulation may be associated with increased delivery of EPA.

### 3.4. EPA-Liposomes Inhibited Production of NO and ROS, and Cytokine Secretion in Activated HL60 Cells

**Figure 5:**
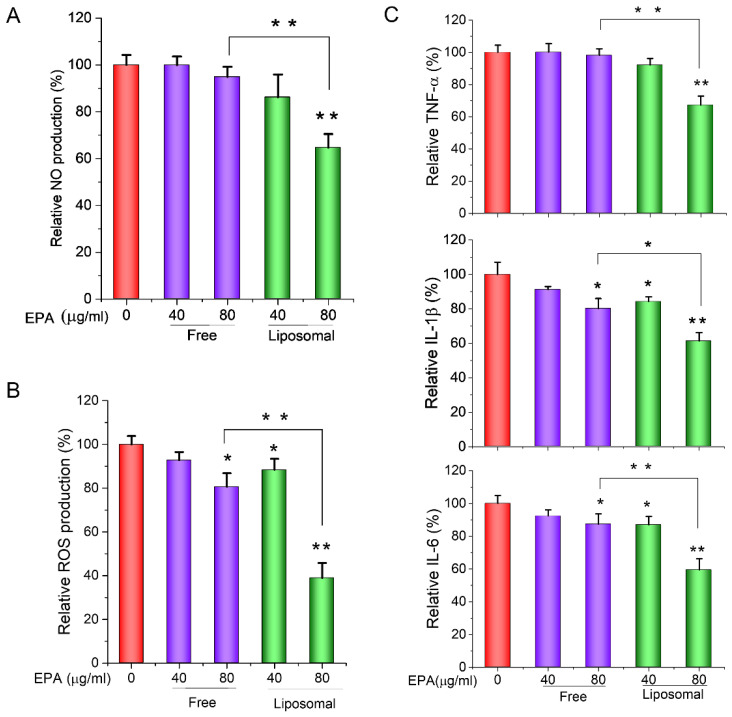
The inhibition of production of NO, ROS, and cytokines by EPA-liposomes. The differentiated HL60 cells were treated with free EPA or EPA-liposomes at the indicated concentrations in the presence of LPS (100 ng/mL) for 24 h, and the NO contents (**A**) in the cell culture media were determined by the Griess agent. (**B**) The ROS levels in the cells were determined by the ROS detection kit. (**C**) The cytokine levels in the cell culture medium were determined by the ELISA kit following the guidance of the manual. The data are expressed as means ± SD. * *p* < 0.05, ** *p* < 0.01, *n* = 3.

Unbalanced NO and ROS are often secreted by activated immune cells and play a key role in the development and progression of inflammatory diseases [23,24,25]. It was reported that neutrophils, along with macrophages, are a crucial source of ROS [26]. Therefore, the NO and ROS levels in neutrophils were determined, respectively. We studied the production of NO, ROS, and cytokines, including TNF-α, IL-1β, and IL-6, and found upon stimulation by 100 ng/mL LPS, HL60 cells produced higher levels of NO (Figure 5A), ROS (Figure 5B), and cytokines (Figure 5C). However, when liposomal EPA (40 µg/mL and 80 µg/mL) was used, the production of NO, ROS and cytokines was significantly inhibited. Especially, the liposomal formulation sensitizes HL60 cells to EPA, indicating that a lower concentration (40 µg/mL) of liposomal EPA could also suppress the production of the above inflammatory indicators (Figure 5).

### 3.5. The Liposomal Formulation Increased the Conversion of EPA in Activated HL60 Cells

**Figure 6:**
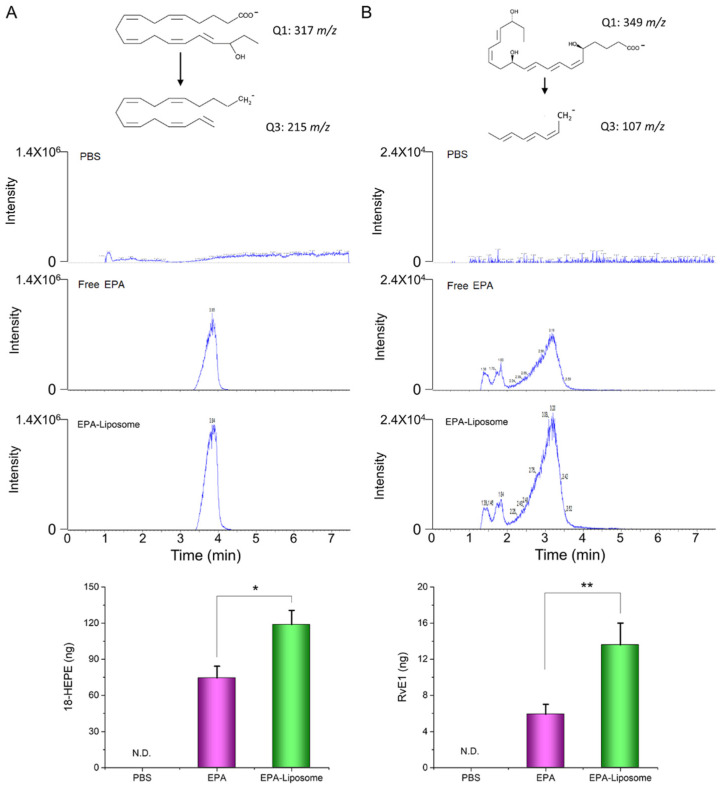
LC-mass spectrometry determination of the metabolites of EPA. The differentiated HL60 cells were incubated with EPA in the form of either free form or liposomal formulation for 8 h. The metabolized 18-HEPE (**A**) and RvE1 (**B**) in the cell culture medium were extracted with chloroform after the proteins were removed by 70% ice ethanol. N.D., not detectable. The data are expressed as means ± SD. * *p* < 0.05, ** *p* < 0.01, *n* = 3.

The previous studies have shown that the metabolites of EPA, such as RvE1, possess the anti-inflammatory effect on various of inflammation disease [27,28,29]. To elucidate the anti-inflammatory mechanism of EPA, we measured the contents of 18-HEPE and RvE1, the EPA-derived pro-resolving mediators, in the cell culture medium after incubation. It was found that although free EPA can be metabolized by the cells, the liposomal formulation can enhance the production of 18-HEPE (Figure 6). As shown in Figure 6A, the EPA-liposome group synthesized around 1.5-fold of 18-HEPE compared to the free EPA group, while almost 2-fold of RvE1 was produced in the liposomal EPA group compared to the free EPA group (Figure 6B). The results suggest that liposomal formulations enhanced the production of RvE1 because of increased uptake of EPA. Furthermore, RvE1 binds to GPCR receptors [30,31], then inhibiting the production of NO, ROS, and cytokines (as shown in Figure 5).

We have demonstrated that EPA-loaded liposomes can be efficiently internalized by neutrophil-like cells (HL 60 cells), and the loaded EPA was released gradually in the cells, facilitating the conversion to RvE1 by the cytoplasmic 5-lipoxygenase (LOX) and 15-LOX, two enzymes necessary for PUPA metabolism [32]. The synthesized resolving mediators may bind to the receptor of RvE1 to inhibit the production of NOs, ROS, and cytokines from HL 60 cells.

The primary goal of this study is to understand whether liposomes enhanced the production of RvE1 from EPA, so we performed in vitro studies. The physiological environment is different from the in vitro conditions; thus, the observed phenomenon is required to confirm in vivo in the future. However, recent studies show that PEGylation may selectively enhance the phagocytosis of liposomes by human neutrophils [33]. Therefore, PEGylated liposomes could serve as an appropriate carrier for EPA delivery, and we may expect the promising outcomes of our formulations in vivo.

## 4. Conclusions

In summary, EPA can be loaded into liposome formulations, and the resulting EPA-liposomes can exert a better anti-inflammatory effect by enhancing EPA delivery in HL 60 cells. The mechanism of the enhanced effect is due to the enhanced synthesis of the pro-resolving mediators by the liposome formulation. The EPA-loaded liposomes may serve as potential therapeutics for inflammatory disease therapy.

## Data Availability

The data presented in this study are available on request from the corresponding author.
